# Compassion is a key quality for palliative care teams

**DOI:** 10.1002/cam4.1804

**Published:** 2018-10-10

**Authors:** Jung Hun Kang, Se Il Go, Eduardo Bruera

**Affiliations:** ^1^ School of Medicine Gyeongsang National University Jinju Korea; ^2^ Department of Internal Medicine Gyeongsang University Hospital Jinju Korea; ^3^ Department of Internal Medicine Gyeongsang National University Hospital Changwon Korea; ^4^ Department of Palliative Care and Rehabilitation Medicine University of Texas M.D. Anderson Cancer Center Houston Texas

## Abstract

Multidisciplinary team members including doctors, nurses, and chaplains, and social workers involve palliative. Therefore, it is not easy to define palliative care in a word. We are convinced that compassion is at the core of palliative care.

Kim, a 60‐year‐old man, visited the emergency room because of fever. He was diagnosed with metastatic large cell neuroendocrine carcinoma involving the liver 6 months ago and received etoposide/cisplatin chemotherapy 7 days before this visit. He and his wife, the main caregiver, had been struggling with the treatments for 6 months in the hospital 400 km away from their home. He complained of right upper quadrant pain, decreased appetite, and general weakness. His body temperature was 38.2°C, and his heart rate was 115 bpm. A slight tap on the right upper quadrant of his abdomen caused severe pain. He was hospitalized, and antibiotics were administered under the presumption he had a biliary tract infection. Under the provision of supportive care and compassionate counseling by his physician, Kim got much better in a week.

Before discharge, Kim and his wife talked about how different their impressions of the two hospitals were. “When we visited the previous hospital for chemotherapy, we were given a professional and reasonable explanation by the doctor at the clinic. After hearing the explanation, we had no choice but to rely on him because we didn't know what else we could do. But he felt too far away and difficult for me. It was as if he was trying to keep a distance between him and us.”

Ever since I was hospitalized in this hospital, I could tell that you were treating me with a sincere heart. You seemed to be genuinely concerned about my condition, and that simple fact relieved me so much.

Medical practice is performed amid contrasting issues: scientific knowledge about diseases and subjective emotion; certainty based on the evidence and uncertainty for effectiveness of surgical or medical intervention; unrealistic hope and painful truth; curative treatment and care.^1^ However, these seemingly antithetic pairs are more like complementary concepts. These issues exist at the same time, not sequentially or separately.

Cancer patients need curative services and supportive care simultaneously.^2^ To be more specific, medical services for cancer patients consist of palliative care and curative treatments such as chemotherapy and surgery. Patients are not satisfied with either anticancer treatment alone or palliative care by itself. The integration of appropriate anticancer therapies and physician support is what perfects the quality of medical treatment provided. It is important to note that the proportion of these two components changes over the course of cancer.^3^ When a cure is possible, all patients, family members, and physicians should focus on curative treatments. However, as cancer progresses, the hope of improvement often fades away, and the limitations of achievable goals become clear. For example, although many promising anticancer drugs, including immune checkpoint inhibitors, give unprecedented hope to cancer patients and their families, they barely manage to extend life, let alone the hope of a complete cure. Palliative care should be reinforced in this case because the role of curative treatment is reduced. Ultimately, when cancer patients reach the end of their lives, palliative care, including hospice, accounts for the majority of medical services provided.

What qualities are required to become a physician skilled at curative treatment? There does not seem to be much room for debate on this matter; a wealth of knowledge, experience, and skill will suffice. Then, what qualifications are necessary for palliative care? Of countless virtues, compassion would unquestionably rank the highest. Patients prefer physicians with compassion the most,^4^ and Teno et al insisted that compassionate palliative care teams help to ensure high‐quality palliative care.^5^ Compassionate staff naturally try to address the suffering and needs of their patients through relational understanding.^6^

Therefore, compassion can be considered an essential quality for palliative care teams (PCTs) including nurses, palliative physicians, chaplains, and even hospice volunteers.^7^, ^8^ PCTs should have the ability to listen, empathize and maintain solidarity with patients to their satisfaction.^9^ PCT members with sincere empathy naturally honor cultural traditions and treat their patients accordingly and respectfully. Medical personnel who respect patients for themselves are bound to meet these mentioned conditions.

While it is very difficult for patients to assess therapeutic qualities, such as professional knowledge and skill, they are extremely keen on assessing emotional qualities such as compassion, even subtle acts, such as the physician's posture or eye contact. Bruera et al reported that patients showed a preference toward physicians who took a sitting rather than a standing posture during the inpatient consultation, perceiving them as more compassionate.^10^ Another study revealed that patients felt that physicians who practiced in a face‐to‐face manner were more compassionate than professionals with a computer in the examination room during the medical encounter.^11^ The same group reported that patients’ perceptions of physician compassion are not affected by clinical outcome but rather by the optimism of their clinical message.^12^ Based on previous studies, patients are likely to extrapolate therapeutic professionalism and trustworthiness from the amount of compassion provided by physicians. Compassion also allows the healthcare providers to treat patients suffering without any prejudice.

However, the continuum of compassionate care may also lead to the depletion of the spiritual energy of a PCT. Therefore, they need effective and personalized recesses, including meditation, religious activity, or quality time with family, to restore their compassionate energy. The formation of compassion is not simply stem from the inherent elements of physician. It is significantly influenced by another various aspects such as environmental, patient, systemic, and clinical factors. Thus, comprehensively addressing these issues are also required to minimize compassion fatigue.^13^ Otherwise, compassion fatigue leads to the emotional exhaustion of PCTs and burned out PCTs may treat patients in an impersonal manner and even demonstrate cynical behavior.

In summary, medicine consists of curative and care services. The ratio varies depending on the medical situation; care services should mainly be considered when there is no hope for improvement of the disease itself. Compassion is the key quality for healthcare providers who are involved in care services. Thus, PCT members should treat their patients with profound compassion and at the same time develop their own stress outlets to prevent burnout.

## References

[1] Clark SB . Mark twain and medicine. N Engl J Med. 2004;350:2529‐2530.

[2] Armes J , Crowe M , Colbourne L , et al. Patients' supportive care needs beyond the end of cancer treatment: a prospective, longitudinal survey. J Clin Oncol. 2009;27:6172‐6179. 19884548 10.1200/JCO.2009.22.5151

[3] Ferris FD , Bruera E , Cherny N , et al. Palliative cancer care a decade later: accomplishments, the need, next steps—from the American Society of Clinical Oncology. J Clin Oncol. 2009;27:3052‐3058. 19451437 10.1200/JCO.2008.20.1558

[4] Sinclair S , Beamer K , Hack TF , et al. Sympathy, empathy, and compassion: a grounded theory study of palliative care patients’ understandings, experiences, and preferences. Palliat Med. 2017;31(5):437‐447. 27535319 10.1177/0269216316663499 PMC5405806

[5] Teno JM , Connor SR . Referring a patient and family to high‐quality palliative care at the close of life: "We met a new personality.. with this level of compassion and empathy". JAMA. 2009;301:651‐659. 19211472 10.1001/jama.2009.109

[6] Sinclair S , McClement S , Raffin‐Bouchal S , et al. Compassion in health care: an empirical model. J Pain Symptom Manage. 2016;51(2):193‐203. 26514716 10.1016/j.jpainsymman.2015.10.009

[7] Schantz ML . Compassion: a concept analysis. Nurs Forum. 2007;42:48‐55. 10.1111/j.1744-6198.2007.00067.x 17474937

[8] Compassion is key for hospice volunteer; 2017 https://www.mankatofreepress.com/news/local_news/compassion-is-key-for-hospice-volunteer/article_49ea1c60-fd32-11e6-86ff-9f6be48dbd2b.html. Accessed June 6, 2018.

[9] Saunders J . The practice of clinical medicine as an art and as a science. Med Humanit. 2000;26:18‐22. 12484313 10.1136/mh.26.1.18

[10] Strasser F , Palmer JL , Willey J , et al. Impact of physician sitting versus standing during inpatient oncology consultations: patients' preference and perception of compassion and duration. A randomized controlled trial. J Pain Symptom Manage. 2005;29:489‐497. 15904751 10.1016/j.jpainsymman.2004.08.011

[11] Haider A , Tanco K , Epner M , et al. Physicians' compassion, communication skills, and professionalism with and without Physicians' use of an examination room computer: a randomized clinical trial. JAMA Oncol. 2018;4(6):879‐881. 29710136 10.1001/jamaoncol.2018.0343 PMC6584321

[12] Tanco K , Azhar A , Rhondali W , et al. The effect of message content and clinical outcome on patients' perception of physician compassion: a randomized controlled trial. The Oncologist. 2018;23(3):375‐382. 29118266 10.1634/theoncologist.2017-0326 PMC5905680

[13] Fernando AT , Arroll B , Consedine NS . Enhancing compassion in general practice: it’s not all about the doctor. Br J Gen Pract. 2016;66:340‐341. 27364652 10.3399/bjgp16X685741 PMC4917021

